# NTRK1 Gene Fusions Are Frequent in Juvenile Xanthogranuloma

**DOI:** 10.1097/PAS.0000000000002405

**Published:** 2025-04-16

**Authors:** Elisabeth Schlögl, Helga Hürner-Unterberger, Ingrid Simonitsch-Klupp, Gabriele Amann, Jaqueline Blank-Foltin, Barbara Neudert, Lisa Wozelka-Oltjan, Christine Haberler, Georg Ebetsberger-Dachs, Leonhard Müllauer

**Affiliations:** * Department of Pathology; § Division of Neuropathology and Neurochemistry, Department of Neurology, Medical University of Vienna; † Division of Hematology and Oncology, Department of Internal Medicine III, Klinik Favoriten, Vienna; ‡ Department of Paediatrics and Adolescent Medicine, Johannes Kepler University Linz, Kepler University Hospital, Linz, Austria

**Keywords:** non-Langerhans cell histiocytosis, juvenile xanthogranuloma, NTRK1, targeted therapy, molecular pathology

## Abstract

Juvenile Xanthogranuloma (JXG) is a rare form of non-Langerhans cell histiocytosis. The most common known gene mutations affect the mitogen-activated protein (MAP) kinase, phosphoinositide 3-kinase (PI3K), and Janus kinase/signal transducer and activator of transcription (*JAK*/*STAT*) signaling pathways. We present a case of congenital JXG in a premature newborn from a dicygotic twin pregnancy with subdermal infiltrates on the chest, hepatosplenomegaly, ascites, pancytopenia, and petechiae on the abdomen and extremities. Next-generation sequencing of tissue from a subdermal infiltrate revealed a tropomyosin 3::neurotrophic tyrosine kinase receptor (*TPM3*::*NTRK1*) gene fusion. Therefore, a retrospective analysis of 34 additional non-Langerhans cell histiocytoses (16 JXG, 3 adult xanthogranuloma and 1 benign cephalic histiocytosis, both clinical subtypes of JXG, as well as 13 Rosai-Dorfman and 1 Erdheim-Chester disease) for *NTRK*1, 2 and 3 aberrations was performed. This analysis revealed an *NTRK1* gene fusion in 4 additional JXGs and 1 adult xanthogranuloma. In conclusion, NTRK1 gene fusions are moderately common in JXG (6/21; 28.6% in our series). This finding places JXG in the category of proliferative diseases with one of the highest frequencies of *NTRK* gene rearrangements. Therefore, *NTRK* gene fusions should be included in a gene panel test for difficult-to-treat JXG. Given the potential of NTRK gene fusions as a therapeutic target, *NTRK* inhibitors may represent a novel effective treatment for JXG with a challenging clinical course.

Juvenile Xanthogranuloma (JXG) is a rare proliferation of non-Langerhans cell histiocytes. It occurs primarily in young children. More than 70% occur in the first year of life, and 20% to 35% are congenital.^1^^–^^6^ It typically affects the skin with asymptomatic single or multiple reddish or yellowish papules and nodules, most commonly on the head, neck, and trunk. The lesions usually resolve spontaneously within months or years, sometimes leaving minimal skin atrophy or a hyperpigmented scar.

The disease may also be localized to single extracutaneous sites or be systemic in up to 4% of cases.^6^^,^^7^ Systemic JXG presents with multiple skin lesions and visceral, brain, and bone marrow infiltrates. Although it also resolves spontaneously in most cases, it can progress without therapy and be life-threatening.^8^ Treatment regimens for systemic JXG are those for Langerhans cell histiocytosis (LCH), including chemotherapeutic agents such as antimetabolites or vinca-alcaloids.^9^ A protocol with clofarabine, cladribine, or cytarabine has been used in relapsed or refractory systemic JXG with variable response.^10^ These chemotherapeutic agents are nonspecific and have a broad spectrum of potential adverse events.

JXG shares with LCH and other non-LCH disorders, such as Rosai-Dorfman and Erdheim-Chester, common somatic mutations in mitogen-activated protein (MAP) kinase, phosphoinositide 3-kinase (PI3K), and Janus kinase/signal transducer and activator of transcription (JAK/STAT) signaling pathway genes such as *CSF1R*, *KRAS*, *MAP2K1,* and *NRAS*.^11^^–^^14^ In addition, a germline *NF1* mutation drives the development of JXG in ~30% of children with neurofibromatosis 1 under the age of 2.^15^ Therefore, targeting mutant proteins in the above signaling pathways is an emerging alternative option for those who require systemic treatment.

We diagnosed and treated a newborn (preterm at 30 + 1 wk of gestation) from a dicygotic twin pregnancy with congenital JXG. RNA gene panel sequencing of a skin infiltrate revealed the presence of a tropomyosin 3-neurotrophic tyrosine kinase receptor (*TPM3*::*NTRK1*) gene fusion. The *NTRK1* and related *NTRK2* and *NTRK3* genes encode the tropomyosin receptor kinase (TRK) receptors Trk A, B, and C, respectively.^16^ Overall, *NTRK* 1,2,3 gene fusions are very rare, occurring in only 0.2% to 0.3% of all malignancies.^17^^–^^19^ However, in a very small number of neoplasms, *NTRK* 1,2,3 fusions are common and in some even pathognomic. These very rare neoplasms include infantile fibrosarcoma, mesoblastic nephroma, and mammary analog secretory carcinoma of the salivary gland and breast.^20^^,^^21^ *NTRK* gene fusions are a therapeutic target, and TRK inhibitors such as larotrectinib and entrectinib have been approved.^22^ *NTRK* fusions in JXG have been previously described in a few case reports and in a larger study of genetic alterations in histiocytosis.^12^^,^^13^^,^^23^^–^^27^

Inspired by our patient with congenital JXG we wanted to determine the frequency of *NTRK* gene fusions in non-LCHs, particularly JXG. We expected that the results would contribute to a better understanding of the pathogenesis of non-LCH. Furthermore, a deeper knowledge of the nature and frequency of *NTRK* gene fusions in non-LCH may provide a basis for future molecular diagnostic and therapeutic approaches in non-LCH.

## MATERIALS AND METHODS

### Ethical Approval

The study was approved by the Ethics Committee of the Medical University of Vienna (vote number 1301/2021).

### Cases

A formalin-fixed, paraffin-embedded (FFPE) tissue block of a subcutaneous thoracic tumor was received from an external hospital for histopathologic consultation. The tumor had developed in a newborn of a dicygotic twin pregnancy. The diagnosis was non-Langerhans cell histiocytosis (non-LCH), most likely of the xanthogranuloma family (disseminated xanthogranuloma or juvenile xanthogranuloma). RNA sequencing revealed the presence of a *TRPM*::*NTRK1* fusion transcript, and immunohistochemistry confirmed TRK protein positivity of lesional cells. In addition, fluorescence in situ hybridization (FISH) revealed the presence of an *NTRK1* gene rearrangement.

To address the question of whether *NTRK* gene fusions are a recurrent genetic aberration in non-LCH, we identified 34 additional cases of non-LCH with paraffin-embedded tissue (n=28) or archived unstained slides (n=6) available for immunohistochemistry and/or mutation testing. Cases were diagnosed at the Institute of Pathology, Medical University of Vienna, between 2000 and 2021. The cases were classified as follows: 16 cases of juvenile xanthogranuloma (JXG; mean patient age 2.7 y), 3 cases of adult xanthogranuloma and 1 case of benign cephalic histiocytosis. Adult xanthogranuloma and benign cephalic histiocytosis are clinical variants of JXG. 13 cases of Rosai-Dorfman disease and 1 case of Erdheim-Chester disease were also studied.

### Tissue Microarray

A tissue microarray (TMA) was generated utilizing a TMA grandmaster instrument (3D Histotech). As the majority of tumors exhibited a spatially homogeneous morphology, a 2 mm core was extracted from each of the collected FFPE samples (n=26). Three cases exhibited 2 morphologically distinct tumor components. In these cases, one core was taken from each region. The obtained cores were embedded in a paraffin block and histologic sections were stained with hematoxylin and eosin for morphologic correlation and with a pan-tropomyosin receptor kinase (TRK) immunohistochemical assay (Ventana Medical Systems).

### Immunohistochemistry

Archived unstained whole tissue sections for samples without an available FFPE tissue block (n=6), fresh FFPE tissue sections (n=3), and the TMA (n=26), including the index case, were stained for TRK A, B, and C expression using a pan-TRK rabbit monoclonal antibody (clone EPR17341; Ventana Medical Systems) on a Benchmark Ultra stainer (Ventana Medical Systems). In this study, positivity was defined as the presence of membranous, cytoplasmic, or nuclear staining. Two *NTRK* wild-type JXGs with a pathogenic/likely pathogenic MSH2 and PMS2 DNA sequence variant were additionally stained for the expression of MLH1, MSH2, MSH6, and PMS2 to verify the presence of mismatch repair protein loss. The antibodies used were MLH1 (clone M1, Ventana Medical Systems), MSH2 (clone G219-1129), MSH6 (clone 44), and PMS2 (clone EPR3947). The last 3 antibodies were provided by Cell Marque, Rocklin, CA.

### Fluorescence In Situ Hybridization (FISH)

Fluorescence in situ hybridization (FISH) was performed using 4 μm thick FFPE tissue sections (cases 1 and 5 in Table 1), a 4 μm thick TMA section, and archived unstained slides in 6 cases where FFPE tissue blocks were not available. The *NTRK1* and *NTRK3* break-apart FISH probes were used (Zytovision). A total of 100 nuclei per tumor were analyzed. A break-apart signal in ≥15% of the nuclei was considered aberrant for both *NTRK1* and *NTRK3* FISH.

**TABLE 1:** Clinical and Molecular Features of Xanthogranulomas With NTRK1 Gene Rearrangement

Case	Diagnosis	Age	Sex	Tumor localization	Pan-TRK IHC	NTRK fusion/rearrangement	Other mutations
1 Index case	JXG	20 d	M	Subcutaneous, thoracal	Positive	TPM3 (exon 7)::NTRK1 (exon 10)	None
2	JXG	4 y	M	Eyelid	Positive	TPM3 (exon 7)::NTRK1 (exon12)	Not done*
3	JXG	2 y	F	Skin, axilla	Positive	IRF2BP2 (exon 1)::NTRK1 (exon12)	None
4	JXG	15 mo	M	Upper lip	Positive	TPM3 (exon 7)::NTRK1 (exon12)	DNA sequencing failed
5	AXG	47 y	M	External acoustic meatus	Positive	NTRK1 rearrangement†, fusion partner unknown	None
6	JXG	17 mo	f	Left shoulder	Uncertain	IRF2BP2 (exon 1)::NTRK1 (exon 10)	None

* No tissue available for DNA sequencing.

† Fluorescence in situ hybridization (FISH) result, RNA sequencing failed.

AXG indicates adult xanthogranuloma; JXG, juvenile xanthogranuloma.

### Targeted Next-generation Sequencing

DNA and RNA were isolated from FFPE tissue. DNA was extracted using the Maxwell FFPE Plus DNA Kit (Promega), whereas RNA was extracted using the Maxwell RSC RNA FFPE Kit (Promega) on a Maxwell RSC instrument (Promega). DNA concentrations were quantified using the Qubit dsDNA HS Assay Kit (Thermo Fisher Scientific) on a Qubit 2.0 Fluorometer (Thermo Fisher Scientific). RNA concentrations were determined using the QuantiFlour RNA System (Promega) on a Quantus Fluorometer (Promega). A total of 40 ng of RNA was transcribed into cDNA using the Vilo enzyme (SuperScript Vilo, Thermo Fisher Scientific). Next-generation sequencing (NGS) was performed using the Oncomine Comprehensive Assay v3 (Thermo Fisher Scientific). The gene panel includes 161 genes by DNA sequencing and 51 genes by parallel RNA sequencing. Libraries were loaded onto sequencing chips using an Ion Chef instrument (Thermo Fisher Scientific) and sequenced on an Ion S5 system (Thermo Fisher). The generated sequencing data were analyzed using Torrent Suite and Ion Reporter software (Thermo Fisher Scientific). The Franklin platform from Genoox (https://franklin.genoox.com) and VarSome (https://varsome.com/) were also used for sequence variant interpretation.^28^

## RESULTS

**FIGURE 1:**
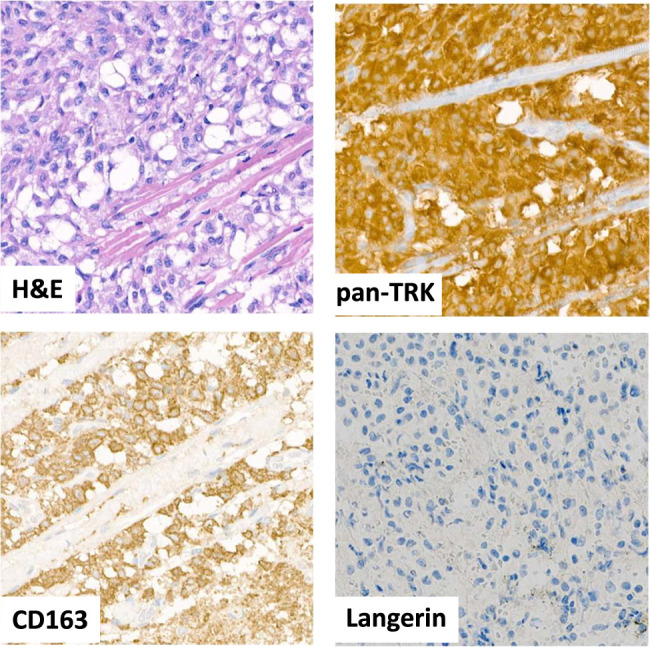
Hematoxylin-eosin (H&E) and immunohistochemical staining of the thoracic subcutaneous JXG infiltrate of the index case (case 1 in Table 1).

A *TPM3* (exon 7)::*NTRK1* (exon 10) gene fusion was identified in a congenital juvenile xanthogranuloma of a male newborn from a dizygotic twin pregnancy. The infant was born at 30 + 1 weeks of gestation with a birth weight of 1528 g. During the initial period of life, the infant developed a series of symptoms, including ascites, pancytopenia, immunoglobulin deficiency, impaired hepatic synthesis with hypoalbuminemia, and pathologic coagulation with petechiae on the abdomen and extremities. During the first 7 days after birth, 2 subdermal masses of increasing size were observed on the dorsal aspect of the thorax and the ventral aspect of the costal arch. A biopsy was taken from the dorsal tumor. Histologic examination revealed the presence of adipose and muscle tissue, accompanied by a diffuse and dense infiltration of small to medium-sized cells. The nuclei were irregularly shaped and often situated in an eccentric position, with a folded or kidney-shaped morphology. The cytoplasm was predominantly wide, foamy, and frequently vacuolated. Neither eosinophil granulocytes nor touton-like giant cells were identified. Immunohistochemical staining showed positive reactivity for CD14, CD33, CD45, CD68R, CD163, and factor XIIIa (Fig. 1). The S100 reactivity was inconclusive, with positive staining observed in only a few areas. The following markers were negative: CD1a, CD10, CD30, CD34, CD123, ALK, alpha-actin, BRAF V600E, desmin, EMA, Langerin, myeloperoxidase, and tryptase. Pan-TRK A, B, and C staining was positive, and subsequent RNA gene panel sequencing revealed a *TPM3* (exon 7)::*NTRK1* (exon 10) fusion transcript. The diagnosis was non-LCH, most likely of the xanthogranuloma family (disseminated xanthogranuloma or JXG).

As the patient’s clinical condition worsened, with arterial hypotension, hepatomegaly, anuria, and elevated inflammatory markers, treatment with dexamethasone (10 mg/m² BSA) was initiated according to the 2004 treatment regimen for HLH. After the administration of dexamethasone, the child’s condition rapidly improved and the dermal infiltrates regressed, so the dexamethasone was tapered and discontinued after one month. Now, at the age of 6, he is doing well, with a slight developmental delay and mild muscle hypotonia. His twin sister is also doing well.

Given the paucity of previously reported non-LCH cases with NTRK rearrangements, we performed a retrospective analysis of 34 additional non-LCH cases to determine the prevalence of NTRK gene fusions. A tissue microarray (TMA) from 26 cases, including the index case, was stained with a pan-TRK antibody. In addition, whole tissue sections from 3 cases were stained with the same antibody. Immunohistochemistry from 6 cases (4 juvenile xanthogranulomas, 1 benign cephalic histiocytosis, and 1 Rosai-Dorfman disease) with only archived unstained tissue sections was negative, but was considered unreliable due to the potential loss of antigenicity that can occur in unstained slides archived for extended periods at room temperature. Therefore, these cases were further evaluated by fluorescence in situ hybridization (FISH) to determine the presence of NTRK1 and NTRK3 rearrangements.

Five cases, including the index case, showed a positive immunohistochemical reaction on TMA. Notably, the pan-TRK-positive tumors included only xanthogranulomas (4 juvenile and 1 adult xanthogranuloma) and not other non-LCH cases (thirteen Rosai-Dorfman and one Erdheim-Chester disease). Pan-TRK immunohistochemical staining showed a relatively consistent pattern in all 5 positive cases, with diffuse reactivity observed in the cytoplasm. However, there was a notable focal increase in staining intensity along the cell membrane (Fig. 1).

### Fluorescence In Situ Hybridization (FISH)

**FIGURE 2:**
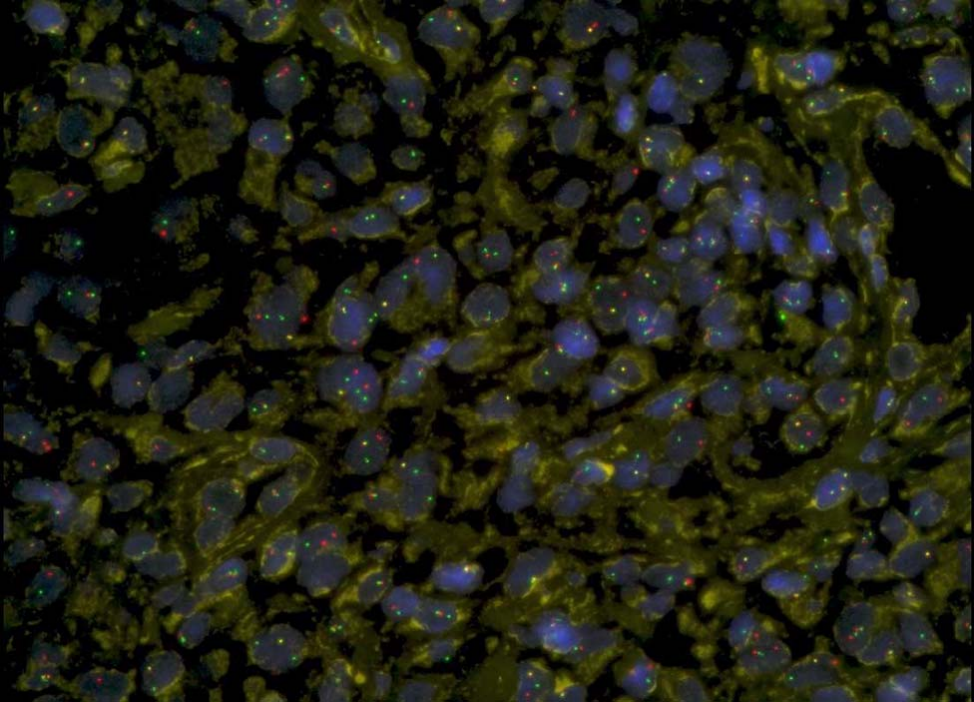
Fluorescence in situ hybridization (FISH) with an *NTRK1* gene break-apart probe of tissue from the index case (case 1 in Table 1).

The 5 samples with pan-TRK immunoreactivity from the TMA showed NTRK1 break-apart signals in FISH, thereby confirming the presence of an *NTRK1* gene rearrangement (Fig. 2). They did not show an additional NTRK3 gene rearrangement. On the contrary, all the other 27 non-LCH samples analyzed by FISH showed normal fluorescence signals of the *NTRK1* and *NTRK3* genes. The *NTRK2* gene was not examined by FISH, but NTRK2 gene fusions are reported to be even much rarer than *NTRK1* and *NTRK3* gene rearrangements in other neoplastic diseases.

### Targeted Next-generation Sequencing

DNA gene panel sequencing was performed on 28 tissue samples (13 JXG, 3 AXG, 11 RDD, 1 ECD) and RNA gene panel sequencing was also performed on 29 tissue samples (14 JXG, 3 AXG, 11 RDD, 1 ECD). Diagnostically useful DNA sequencing results were obtained in 20 of the samples (10 JXG, 3 AXG, 7 RDD) and RNA sequencing results were obtained in 12 of the samples (7 JXG, 1 AXG, 4 RDD). The high failure rate, especially for RNA sequencing, was most likely due to the advanced age of the FFPE blocks, which ranged from 10 days to 24 years (median 9.0 y, average 9.37 y). No pathogenic/likely pathogenic DNA mutation was detected in 14 cases, (8 JXG, 2 AXG, 4 RDD). Pathogenic/likely pathogenic mutations were detected in 6 cases (2 JXG, 1 AXG, 3 RDD). These included the genes *ATR*, *NRAS*, *MAP2K1*, *TSC1*, *MSH2,* and *PMS2* (Supplementary Data Table 2, Supplemental Digital Content 2, http://links.lww.com/PAS/C99). RNA sequencing revealed *NTRK1* in-frame gene fusions in 4 JXG and 1 AXG tissues (Table 1 and Supplementary Data Table 1, Supplemental Digital Content 1, http://links.lww.com/PAS/C98). The *NTRK1* gene fusions were with tropomyosin 3 (*TPM3*) as a partner gene in 3 cases and with interferon regulatory factor 2 binding protein 2 (*IRF2BP2*) in 2 cases (Table 1 and Supplementary Data Table 1, Supplemental Digital Content 1, http://links.lww.com/PAS/C98). One of the 2 IRF2BP2 fusion-positive JXG showed uncertain pan-TRK immunoreactivity on repeated staining (case 6, Table 1). In the case of one AXG with an NTRK1 rearrangement by FISH, RNA sequencing did not yield results (Table 1). In 4 of the 6 xanthogranulomas (5 JXG, 1 AXG) with a proven *NTRK1* gene rearrangement, no pathogenic/likely pathogenic mutations were identified by DNA sequencing. In one case DNA sequencing failed and in one case not enough tissue was available for sequencing (Table 1).

### Clinical Features of Juvenile and Adult Xanthogranulomas with *NTRK1* Gene Fusion

The clinical characteristics of the 6 cases (5 JXG, 1 AXG) with *NTRK1* rearrangements are summarized in Table 1. The male-to-female ratio was 2:1. In our *NTRK1*-negative JXG and AXG cohort the male-to-female ratio was 0.37:1. Patient age ranged from 20 days to 47 years (median 20.5 mo, mean 111.4 mo). The JXG/AXG lesions were located on the skin, eyelid, and lip. The index patient had at least 2 dermal thoracic skin lesions and systemic symptoms. However, only one skin lesion was examined histopathologically. Therefore, it is unclear whether the systemic symptoms were caused by JXG or by other causes. The other 5 patients had solitary lesions and no systemic symptoms.

### Previously Reported Cases of Non-Langerhans Cell Histiocytosis with NTRK Gene Fusion

A few case reports and one large study report rare *NTRK* gene fusions in non-LCH, including JXG and Erdheim-Chester disease (Table 2).^12^^,^^13^^,^^23^^–^^27^ Of note, *NTRK1* is the gene involved in all of these fusions, and JXG seems to be the non-LCH with the highest incidence of NTRK fusions.

**TABLE 2:** Reports of NTRK Gene Fusions in Non-Langerhans Cell Histiocytosis

Reference	Diagnosis	Cases/with NTRK fusion	NTRK fusion genes	Tissue with NTRK fusion
Pinney et al 2016^23^	Generalized eruptive histiocytosis	1/1 case report	LMNA::NTRK1	Skin, disseminated
Diamond et al 2016^12^	ECD*	9(1 JXG, 7 ECD, 1 Non-LCH NOS)/1 (11%)	LMNA::NTRK1	Skin (analyzed tissue), systemic
Durham et al 2019^13^	JXG† ECD RDD‡	55/6 (11%) 100/1 (1%) 17/0 (0%)	in JXG: 3x IRF2BP2::NTRK1; 1x TPM3::NTRK1; 1x SQSTM1::NTRK1; in ECD: 1x LMNA::NTRK1	Skin in JXG (clinical presentation not reported), soft tissue and bone in ECD
Chan et al 2020^24^	Progressive nodular histiocytosis/xanthogranulomatosis	1/1 case report	IRF2BP2::NTRK1	Skin, disseminated
Ak et al 2022^25^	AXG§	1/1 case report	LMNA::NTRK1	Skin, multifocal
Umphress et al 2023^26^	1x AXG, 1x JXG	4/2 (50% of sequenced cases)	TPM3::NTRK1; PRDX1::NTRK1	Skin, localized
Kim et al 2024^27^	JXG	1/1 case report	IRF2BP2::NTRK1	Skin, disseminated

* Erdheim-Chester disease.

† Juvenile xanthogranuloma.

‡ Rosai-Dorfman disease.

§ Adult xanthogranuloma.

## DISCUSSION

In this study, we found a 28.6% incidence of *NTRK1* gene fusions (6/21) in the JXG cohort, whereas no NTRK gene alterations were identified in Rosai-Dorfman and Erdheim-Chester disease, although the total number of cases was limited to 14. This is a surprising molecular finding as *NTRK* fusions are overall very rare in neoplastic diseases (0.2% to 0.3%).^17^^–^^19^ Only in a few extremely rare entities are *NTRK* fusions observed with significant frequency.^20^^,^^21^ JXG was not included in these entities, and only a few cases of non-LCH with *NTRK* fusion have been described.^12^^,^^13^^,^^23^^–^^27^ Our results suggest that JXG is a proliferative disorder with a moderate prevalence of *NTRK1* gene fusions.

There is limited information on the clinical characteristics of NTRK1 rearranged JXG. In our cohort, the male-to-female ratio of NTRK1-positive JXG was 2:1, whereas in the 6 patients described by Durham et al^13^, the ratio was 5:1. When both studies are combined, the male-to-female ratio is 3:1. Including all cases of *NTRK1* rearranged non-Langerhans cell histiocytosis described in the publications cited in Table 2 and our cohort, the male-to-female ratio is 3.75:1. In our *NTRK1*-negative JXG, excluding AXG patients, the male-to-female ratio was 0.5:1, and in the study by Durham and colleagues it was 1.3:1. Previous studies with large JXG data sets reported overall male-to-female ratios of 1.4:1 and 1.3:1.^1^^,^^2^ Although there is a slight male predominance in JXG in general, there seems to be a more pronounced shift towards the male sex in *NTRK1* fusion-positive non-Langerhans cell histiocytosis, although this needs to be verified with more future cases. The reasons for this apparent male predominance are not known. The mean age of the JXG/AXG NTRK1-positive cases in our study was 20.5 months (average 111.4 mo). In the Durham study, the mean age of NTRK1+ JXG patients was similar at 24.25 months (average 52.3 mo). Our index case had at least 2 skin lesions on the trunk and severe systemic symptoms, and all 5 other cases had single lesions and no systemic JXG-associated disease (Table 1). The study by Durham and colleagues only mentions the biopsy site (skin, trunk in 2 cases and skin, head in 4 cases), but no information is given on the clinical presentation of the NTRK1+ patients. However, *NTRK1* fusions in non-LCH may be associated with multiple skin lesions, as shown by single case reports of generalized eruptive histiocytosis and progressive nodular histiocytosis/xanthogranulomatosis, as well as very rare cases of ECD (Table 2). The only other reported *NTRK1* fusion-positive JXG, besides our index case with systemic symptoms, surprisingly also involved a male dicygotic preterm twin infant.^27^ However, it is unclear whether both patients had systemic JXG as only skin biopsies were histologically analyzed. The pan-TRK inhibitor larotrectinib was planned as initial treatment for 1 year in the report by Kim et al.^27^

The *NTRK* gene fusions that do occur in malignant neoplasms are with a wide variety of fusion partner genes. The fusion partner often provides a dimerization domain, whereas the TRK kinase domain is retained in the fusion protein. TPM3, observed in 3 of our fusion genes, is a common fusion partner, particularly for *NTRK1*, and provides a strong promoter and dimerization domain. The *IRF2BP2* gene, observed in 2 of our fusions, contains a coiled-coil domain that mediates dimerization and oligomerization of the proteins. Dimerization of TRK proteins mimics ligand-induced receptor dimerization and activation, resulting in sustained kinase activation. *NTRK* gene fusions represent a target for tumor-agnostic tumor therapy with specific inhibitors such as larotrectinib and entrectinib, which often achieve substantial and durable responses.^29^

In non-LCH, including JXG, gene mutations converge on the MAPK/ERK, PI3K/AKT, and JAK/STAT pathways.^5^^,^^13^^,^^30^ NTRK signaling involves the MAPK/ERK and PI3K/AKT pathways in addition to the PLCy pathway. Targeted treatments are available for some of these mutations, particularly with BRAF and MEK inhibitors.^31^ However, we did not observe other pathogenic mutations, including *MAPK*/*ERK*, *PI3K*/*AKT,* and *JAK*/*STAT* pathway genes, in 4 of our *NTRK1* rearranged juvenile xanthogranulomas for which DNA gene panel sequencing results were available. Durham et al^13^ reported genetic alterations in the *AR*, *FAT1*, *FLT1*, *MAP3K13*, *MSH3*, *NOTCH2*, *NOTCH4*, *MSH3*, *PTPRD,* and *TET2* genes in their 6 *NTRK1* fusion-positive JXGs. However, to the best of our knowledge, none of these DNA sequence variants can currently be classified as pathogenic or likely pathogenic. They are benign/likely benign sequence variants. Only one *NOTCH4* variant and one *FLT1* variant can, according to our assessment, be classified as a variant of unknown significance. Therefore, we conclude that the *NTRK1* fusion-positive JXG in the cohort described by Durham et al^13^ did not harbor any additional pathogenic/likely pathogenic DNA sequence variants. The *NTRK1* fusions in JXG may, therefore, be mutually exclusive with additional oncogenic mutations. The genes *FAT1, FLT1, MAP3K13,* MSH3, *NOTCH2*, *NOTCH4*, *PTRPD,* and *TET2*, included in the study by Durham et al^13^, were not covered by our 161 gene panel assay. The *CSFR1* mutations reported by Durham and colleagues in 5 JXG, 1 RDD, 1ECD, 1 LCH, and 1 histiocytic sarcoma were not present in any of our 20 samples (10 JXG, 3 AXG, 7 RDD) with an interpretable *CSFR1* gene sequencing result. However, we observed pathogenic/likely pathogenic mutations in the genes *ATR*, *NRAS*, *MAP2K1*, *TSC1*, *MSH2,* and *PMS2* in 6 of our non-LCH cases (2 JXG, 1 AXG, 3 RDD). The *MSH2* and *PMS2* DNA mismatch repair gene variants were not associated with an MSH2 or PMS2 protein loss by immunohistochemistry. We therefore conclude that a functional allele was still present in the lesional cells. Durham et al^13^ reported rare microsatellite instability as assessed by whole-exome sequencing in 3/31 cases of histiocytosis. Two of the 3 cases were systemic JXG lesions in 1-year-old monochorionic and diamniotic twin siblings.

Gene fusions have been reported very rarely in non-LCH, including JXG, and include *BRAF*, *NTRK*, *PDGFRA,* and *RET* gene fusions.^5^^,^^13^ Rare *ALK* fusions have also been described, although the WHO classification now recognizes an ALK-positive histiocytosis as a distinct entity.^32^ Interestingly, all reported *NTRK* fusions in non-LCH involve the *NTRK1* gene. To our knowledge, *NTRK2* and *NTRK3* gene fusions have not been reported in non-LCH.

Screening for the presence of an NTRK fusion protein by immunohistochemistry is a feasible approach for extracranial tumors in general.^18^^,^^33^^–^^35^ However, in brain tumors there is an overlap with endogenous physiological expression in neuronal tissue, which makes this approach difficult. Sensitivity and specificity as a surrogate marker for *NTRK* fusion are high, although results are less good for NTRK3 and some entities, particularly sarcoma.^18^^,^^35^ A positive immunohistochemical reaction with anti-TRK antibodies requires confirmation of gene fusion by sequencing. A recent report describes frequent TRK protein expression in xanthogranuloma (23 of 43 cases, 53.5%) and suggests an association with the solitary variant, as all 7 included cases of disseminated disease did not express TRK.^26^ Only 2 of the pan-TRK-reactive cases have been sequenced and shown to harbor an NTRK1 fusion. However, our index case with 2 dermal lesions and presumed systemic JXG as well as some case reports (Table 2) argue against a restriction of *NTRK* gene fusions to solitary xanthogranuloma lesions.

Our study demonstrated a moderate prevalence of *NTRK1* gene fusions in JXG. A broad molecular characterization of *MAPK/ERK, PIK3CA, and JAK/STAT* pathway genes and gene fusions, including *NTRK* fusions, seems advisable in rare JXG cases with severe disseminated skin disease, extracutaneous manifestation with clinical complications and systemic disease. *NTRK* fusion may be a promising therapeutic target in clinically challenging cases. In our patient we did not use an *NTRK*-inhibitor because there was already a profound improvement of the clinical problems and spontaneous remission of the second tumor when we received the result of the *NTRK* fusion in the surgically removed tumor.

## Supplementary Material
