# Supplementary material for: NTRK1 Gene Fusions Are Frequent in Juvenile Xanthogranuloma
Source: Am J Surg Pathol. 2025 Apr 16;49(8):763–9. doi: 10.1097/PAS.0000000000002405 (PMC12258803; doi:10.1097/PAS.0000000000002405)
Supplement: Supplementary file 1 [file pas-49-763-s001.docx]

**Supplementary Data TABLE 1.** Characteristics of NTRK1 gene fusions

| **Case** | **NTRK fusion/rearrangement** | **Fusion breakpoints** | **Reference sequences** | **Total mapped fusion panel reads** | **Number of fusion reads/% of total mapped reads** |
| --- | --- | --- | --- | --- | --- |
| 1 Index case | TPM3 (exon7)::NTRK1 (exon10) | chr1:154142876 - chr1:156844363 | TPM3: NM_001143416  NTRK1: NM_002529.4 | 644275 | 65148/10% |
| 2 | TPM3 (exon7)::NTRK1 (exon12) | chr1:154142876 - chr1:156845312 | TPM3: NM_001143416  NTRK1: NM_002529.4 | 117457 | 3910/3,3% |
| 3 | IRF2BP2 (exon 1)::NTRK1 (exon12) | chr1:234744241 - chr1:156845312 | IRF2BP2: NM_182972.3  NTRK1: NM_002529.4 | 272434 | 76144/27,9% |
| 4 | TPM3 (exon7)::NTRK1 (exon12) | chr1:154142876 - chr1:156845312 | TPM3: NM_001143416  NTRK1: NM_002529.4 | 89541 | 4287/4,8% |
| 5 | NTRK1 rearrangement^1^, fusion partner unknown | n.a. | n.a. | n.a. | n.a. |
| 6 | IRF2BP2 (exon 1)::NTRK1 (exon10) | chr1:234744241 - chr1:156844350 | IRF2BP2: NM_182972.3  NTRK1: NM_002529.4 | 2843849 | 130291/4,6% |

JXG = juvenile xanthogranuloma, AXG = adult xanthogranuloma. ^1^ Fluorescence in situ hybridization (FISH) result, RNA sequencing failed. n.a. = not applicable
