# Supplementary material for: NTRK1 Gene Fusions Are Frequent in Juvenile Xanthogranuloma
Source: Am J Surg Pathol. 2025 Apr 16;49(8):763–9. doi: 10.1097/PAS.0000000000002405 (PMC12258803; doi:10.1097/PAS.0000000000002405)
Supplement: Supplementary file 2 [file pas-49-763-s002.docx]

**Supplementary Data TABLE 2.** Gene mutations in NTRK1 fusion-negative cases

| **Case** | **Diagnosis** | **Age** | **Sex** | **Tumor localization** | **Pathogenic/likely pathogenic DNA sequence variant** | **Reference sequence** | **Allele frequency** |
| --- | --- | --- | --- | --- | --- | --- | --- |
| 7 | JXG | 15 months | m | skin, perianal | PMS2: c.1866_1866delGinsTA p.(Met622IlefsTer6) | NM_000535.7 | 50,4% |
| 8 | JXG | 5 months | f | skin, thoracic | MSH2: c.1570C>T p.(Arg524Cys)  ATR:c.6688-1G>A p.(?) | NM_000251.3  NM_001184.4 | 11,0%  7,4% |
| 9 | AXG | 71 years | f | skin | NRAS: NRAS c.38G>A p.Gly13Asp  TSC1: c.1525C>T p.Arg509Ter | NM_002524.5  NM_000368.5 | 11,6%  10,2% |
| 10 | RDD | 68 years | m | testis | MAP2K1: c.167A>C p.(Gln56Pro) | NM_002755.4 | 5,3% |
| 11 | RDD | 46 years | m | brain | MAP2K1: c.199G>A p.Asp67Asn | NM_002755.4 | 6,2% |
| 12 | RDD | 11 years | f | lymph node | MAP2K1: c.157T>C p.Phe53Leu | NM_002755.4 | 9,7% |

JXG = juvenile xanthogranuloma, AXG = adult xanthogranuloma, RDD = Rosai-Dorfman disease
